# An Investigation of 5-Halogenated *N*-Indolylsulfonyl-2-fluorophenol Derivatives as Aldose Reductase Inhibitors

**DOI:** 10.3390/medicines12030016

**Published:** 2025-06-23

**Authors:** Antonios Kousaxidis, Konstantina-Malamati Kalfagianni, Eleni Seretouli, Ioannis Nicolaou

**Affiliations:** Department of Pharmaceutical Chemistry, School of Pharmacy, Aristotle University of Thessaloniki, 54124 Thessaloniki, Greece; kalfagian@pharm.auth.gr (K.-M.K.); seretouli@pharm.auth.gr (E.S.)

**Keywords:** diabetes mellitus, chronic complications, aldose reductase, inhibitors, 5-substituted indoles, halogens, sulfonyl moiety, 2-fluorophenol, molecular docking

## Abstract

Background/Objectives: Diabetes mellitus is a group of chronic metabolic disorders characterized by persistent hyperglycemia. Aldose reductase, the first enzyme in the polyol pathway, plays a key role in the onset of long-term diabetic complications. Aldose reductase inhibition has been widely established as a potential pharmacotherapeutic approach to prevent and treat diabetes mellitus-related comorbidities. Although several promising aldose reductase inhibitors have been developed over the past few decades, they have failed in clinical trials due to unacceptable pharmacokinetic properties and severe side effects. This paper describes the design, synthesis, and pharmacological evaluation of four novel 5-halogenated *N*-indolylsulfonyl-2-fluorophenol derivatives (**3a**-**d**) as aldose reductase inhibitors. Methods: The design of compounds was based on a previously published lead compound (IIc) developed by our research group to enhance its inhibitory capacity. Compounds **3a**-**d** were screened for their ability to inhibit in vitro partially purified aldose reductase from rat lenses, and their binding modes were investigated through molecular docking. Results: The presence of a sulfonyl linker between indole and o-fluorophenol aromatic rings is mandatory for potent aldose reductase inhibition. The 5-substitution of the indole core with halogens resulted in a slight decrease in the inhibitory power of **3a**-**c** compared to IIc. Among halogens, bromine was the most capable of filling the selectivity pocket through hydrophobic interactions with Thr113 and Phe115 residues. Conclusions: Although our strategy to optimize the inhibitory potency of IIc via inserting halogen atoms in the indole scaffold was not fruitful, aromatic ring halogenation can be still utilized as a promising approach for designing more potent aldose reductase inhibitors.

## 1. Introduction

Diabetes mellitus (DM) is a group of chronic metabolic disorders of the carbohydrate metabolism in which glucose is both underutilized as an energy source and overproduced due to imbalances in mechanisms of gluconeogenesis and glycogenolysis [1]. Persistent hyperglycemia is a hallmark of DM caused by insufficient insulin secretion by pancreatic beta cells, the inability of peripheral tissues to effectively utilize insulin, or a combination of both. These factors give rise to life-threatening complications that develop rapidly (acute hyperglycemic crises), such as diabetic ketoacidosis and the hyperglycemic hyperosmolar state [2], as well as chronic complications that progress over time and cause damage in several tissues. They are generally classified into microvascular and macrovascular complications based on the size of the blood vessels affected. Microvascular complications include neuropathy, nephropathy, retinopathy, encephalopathy, cardiomyopathy, and pulmonary microangiopathy, while ischemic stroke and coronary and peripheral vascular disease comprise macrovascular complications [3,4,5,6,7].

Exogenous insulin administration is a potent treatment for severe hyperglycemia or when adequate management of the glycemic status cannot be achieved despite using different oral hypoglycemic drugs. However, diabetic patients, especially those with type 2 DM, face a high risk of developing fatal long-term complications due to regular fluctuations in their blood glucose levels [8]. Given these challenges, there is an urgent need to recruit novel pharmacotherapies for the effective fight against DM-related comorbidities.

Aldose reductase (ALR2), a cytosolic enzyme classified in the aldo–keto reductase superfamily, plays a fundamental role in glucose metabolism through the polyol pathway, particularly under hyperglycemic conditions. ALR2 catalyzes the NADPH-dependent reduction of aldehydes to their corresponding alcohols, demonstrating a broad substrate specificity among hydrophobic alkanals, alkenals, and hydrophilic aldoses [9]. In the first rate-limiting step of the polyol pathway, ALR2 converts glucose into sorbitol, which is subsequently oxidized to fructose by sorbitol dehydrogenase. While this pathway remains relatively inactive under normoglycemic conditions, it becomes significantly upregulated during hyperglycemia, leading to an increased sorbitol and fructose accumulation within cells and contributing to the pathogenesis of chronic DM complications through osmotic, oxidative, and glycative stress conditions as well as inflammation [10,11,12,13].

It has been established that aldose reductase inhibitors (ARIs) suppress the first step in the polyol pathway, preventing these harmful processes from occurring. Preliminary research on ARIs showed that at least one carboxylate or imide group and an aliphatic moiety, either in a ring form or as a chain, must be present in the structure for effective enzyme inhibitory activity.

**Figure 1:**
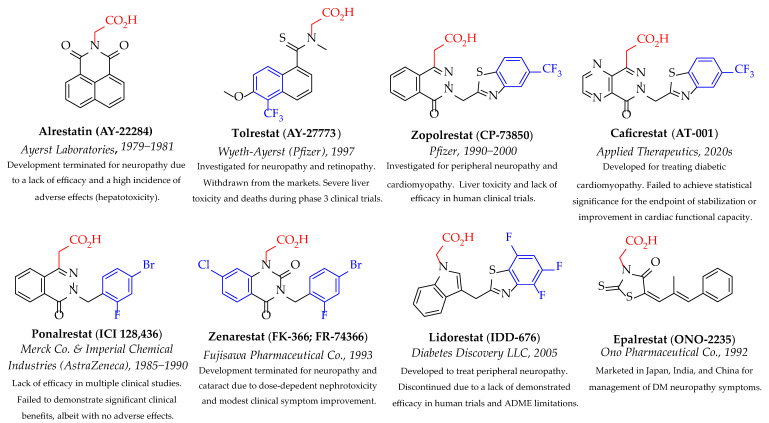
Carboxylic acid derivatives of ARIs reached human clinical trials. Acetic acid pharmacophore and halogenated phenyl moieties are highlighted in red and blue, respectively.

Carboxylic acid derivatives are the most significant and prevalent class of ARI, with acetic acid as their common functional group. Alrestatin, the first ARI established for treating peripheral neuropathy, underwent human clinical trial studies early in the 1980s. However, its clinical development was terminated early due to a high incidence of a photosensitive skin rash, hepatotoxicity, and a lack of efficacy [14]. In later years, more powerful carboxylate ARIs were investigated clinically for treating DM chronic complications, such as tolrestat [15,16], lidorestat [17,18,19], zenarestat [20,21,22], zopolrestat [23,24], ponalrestat [25,26,27,28,29,30], and caficrestat [31]. Nevertheless, they were withdrawn from the late phase of clinical trials due to severe toxicity or disappointing clinical benefits, as illustrated in Figure 1. The only ARI given marketing approval as a therapeutic drug to treat symptoms of diabetic neuropathy is epalrestat; however, it is commercialized only in some Asian countries, like Japan, China, and India [32]. The main drawback of this drug class is its poor pharmacokinetic profile. Carboxylic acid-containing inhibitors demonstrate pKa values of 3–4. Thus, they are highly ionized at the physiological blood pH (7.4), resulting in poor membrane permeability. In addition, they exhibit significant in vivo protein binding and an extended phase II metabolism, which leads to a lack of efficacy and a requirement for particularly high clinical doses [33].

**Figure 2:**
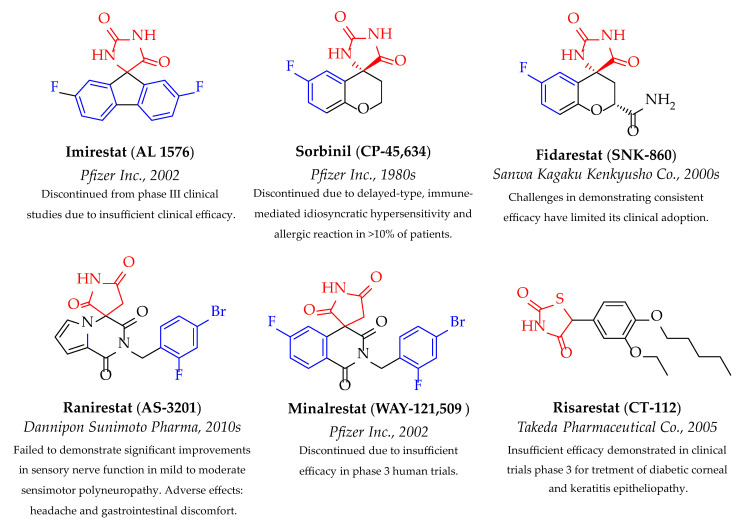
Spirohydantoin, spirosuccinimide, and thiazolidinedione derivatives of ARIs reached human clinical trials. Cyclic imide pharmacophore and halogenated phenyl moieties are highlighted in red and blue, respectively.

The second drug class of ARIs investigated in clinical trials covers mostly spirohydantoin (sorbinil [34,35], fidarestat [36,37], imirestat [38,39], spirosuccinimide (ranirestat [40,41,42], minalrestat [43,44]), and thiazolidinedione (risarestat [45,46,47]) derivatives with a cyclic imide structure as their acidic backbone (Figure 2). Although this drug category showed optimal pharmacokinetic properties and enhanced absorption by target tissues due to higher pKa values (8–9), thus demanding low therapeutic doses, it failed to succeed in human clinical studies due to the exhibition of adverse effects or the inadequate relief of specific disease-related symptoms [48]. Given the failure of these drug classes, there is an urgent need to move forward with new chemotypes of ARIs to prevent long-term diabetic complications.

## 2. Design

**Figure 3:**
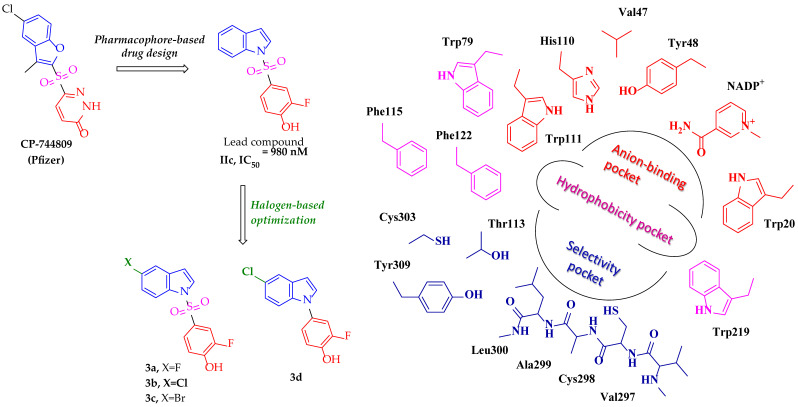
The discovery of compound IIc and the design of potential optimized derivatives **3a**-**d**.

In 2003, Mylari et al. [49] reported the discovery of a novel non-carboxylate, non-spiroimide inhibitor (CP-744809) showing an ideal pharmacokinetic and pharmacodynamic profile in preclinical studies. It demonstrates a sub-nanomolar IC_50_ value (840 pM), an exceptional oral bioavailability (98% at 2 mg/kg), a long plasma half-life (23–29 h), enhanced metabolic stability, and Caco-2 transcellular permeability (>10^−5^ cm/s). CP-744809 demonstrated a remarkable efficiency in vivo in normalizing elevated sorbitol and fructose in the lens, retina, and sciatic nerve of diabetic rats. Inspired by this discovery, our research team recently identified an indole-sulfonyl-o-fluorophenol derivative (IIc) [50], which shares common pharmacophore features with CP-744809 for the potent and selective inhibition of ALR2 (Figure 3). In this design, the 2-fluorophenol moiety was introduced as a bioisosteric replacement for the carboxylic acid group, aiming to preserve interactions with the anionic site of ALR2 while potentially improving the membrane permeability and metabolic stability. Particularly, both compounds include an aromatic ring (pyridazinone or phenyl) targeting the central pocket of the active site, thus forming hydrophobic contacts with the side chains of a tryptophan-rich subpocket (Trp20, Trp79, Trp111, and Trp219) as well as Val47 and Tyr48 residues. This aromatic system incorporates a relatively acidic moiety (acylhydrazone or o-fluorophenol) capable of anchoring the deeply buried substrate-binding pocket, also referred to as an anion-binding pocket, which includes catalytic Tyr48, Lys77, His110, Trp111, and the nicotinamide moiety of NADP^+^. The established hydrogen bonds and electrostatic interactions experienced by these inhibitors are crucial for enzyme inhibition. In addition, the presence of a partly solvent-exposed sulfonyl linker group not only enhances the deprotonation of the head group in the enzyme’s active site but also facilitates the connection of a second aromatic ring (benzofuran or indole) to the selectivity pocket. To fully open this distinct ALR2-specific domain, aromatic rings should form hydrophobic interactions with the side chains of Trp111, Phe122, and Leu300. It has also been proven that aromatic systems can establish π-stacking interactions with the side chain of Trp111 and hydrophobic contacts to Trp79, Phe122, Ala299, and Leu300 residues [48].

It has been previously established that the introduction of halogen atoms into aromatic moieties of ARIs contributes to improving the ligand binding efficiency and pharmacokinetic properties by increasing membrane permeability and reducing undesired ring hydroxylation through the CYP metabolism [51,52]. Moreover, lipophilic hydrogen (F) and halogen (Cl, Br) bond accepting groups at the 5- or p-position of the aromatic system of known ARIs have been recognized as important moieties for penetrating the selectivity pocket and increasing inhibitory power. Based on this rationale, we endeavor to optimize the ALR2 inhibitory capacity of our lead compound by enhancing its lipophilicity via inserting halogen atoms in the 5-position of the indole core. Therefore, we designed and synthesized three halogenated (fluorine, chlorine, and bromine) analogs of IIc (**3a**-**c**) to explore possible interactions (hydrophobic and hydrogen/halogen bonding) between halogen atoms and key distant residues in the ALR2 active site that modulate the conformational opening (Trp111, Phe122, Leu300) and stabilization (Cys80, Thr113, Phe115, Cys303, Tyr309, and Phe311) of the selectivity pocket. Moreover, previous SAR studies on phenyl-sulfonyl-pyridazinone derivatives demonstrated the necessity of the sulfonyl linker for the strong enzyme inhibition [52]. To verify the effectiveness of this spacer in the inhibitory profile of our chemotypes, we further designed and synthesized compound **3d** with a 5-chloro substituent, the most similar analog to CP-744809.

## 3. Materials and Methods

### 3.1. Synthesis

#### 3.1.1. Chemical Reagents and Equipment

All reagents were purchased from Merck SA (Athens) and BLD Pharmatech GmbH (Reinbek, Hamburg, Germany) and used without further purification, except for solvents used for flash chromatography, which were distilled before use. NMR spectra were recorded on an Agilent 500/54 (DD2) spectrometer (500 MHz for ^1^H-NMR, 126 MHz for ^13^C-NMR), and chemical shifts are given in δ referenced to the residual solvent peak. Melting points are uncorrected and determined in open glass capillaries using a Mel-Temp II apparatus. Elemental analyses were performed on a Perkin–Elmer 2400 CHN analyzer. Flash column chromatography was carried out using Merck silica gel 60 (230–400 Mesh ASTM) (Merck SA, Athens, Greece). Elemental analyses were performed by a Perkin–Elmer 2400 CHN analyzer. TLC was run with Fluka Silica gel/TLC-cards. Petroleum ether refers to the fraction with bp 40–60 °C.

#### 3.1.2. Synthesis of Intermediate Compounds **2a**-**c**

To a suspension of 60% NaH in mineral oil (96 mg, 2.4 mmol) in dry tetrahydrofuran (5 mL) was added dropwise a solution of 5-substituted indole **1a**-**c** (1.125 mmol) in dry tetrahydrofuran (5 mL) under a nitrogen atmosphere. The mixture was stirred for 30 min at room temperature, and a solution of the 3-fluoro-4-methoxybenzenesulfonyl chloride (250 mg, 1.112 mmol) in dry tetrahydrofuran (10 mL) was added thereto, and the mixture was stirred at room temperature for 3 h. Thereafter, the solvent was rotary evaporated under reduced pressure. Water (20 mL) and ethyl acetate (20 mL) were added to the residue, and the organic layer was separated, dried, filtered, and then concentrated. The residue was purified by flash column chromatography.

5-fluoro-1-((3-fluoro-4-methoxyphenyl)sulfonyl)-1*H*-indole (**2a**). Eluting system of petroleum ether: ethyl acetate (3:1). Light yellow solid, yield = 72.0%, m.p. = 132–133 °C. ^1^H-NMR (500 MHz, CDCl_3_) δ 7.91 (ddt, *J* = 9.0, 4.3, 0.7 Hz, 1 H), 7.65 (ddd, *J* = 8.7, 2.4, 1.3 Hz, 1 H), 7.58–7.52 (m, 2 H), 7.19 (dd, *J* = 8.7, 2.5 Hz, 1 H), 7.05 (td, *J* = 9.0, 2.5 Hz, 1 H), 6.95 (dd, *J* = 8.7, 7.8 Hz, 1 H), 6.63 (dd, *J* = 3.7, 0.8 Hz, 1 H), 3.88 (s, 3 H). ^13^C-NMR (126 MHz, CDCl_3_) δ 156.54 (d, *J* = 1024.6 Hz), 154.59 (d, *J* = 1033.0 Hz), 152.52 (d, *J* = 6.0 Hz), 150.49, 131.78 (d, *J* = 10.1 Hz), 129.55 (d, *J* = 5.8 Hz), 127.92, 124.29 (d, *J* = 3.8 Hz), 114.86 (d, *J* = 21.8 Hz), 114.47 (d, *J* = 9.5 Hz), 112.94 (d, *J* = 2.0 Hz), 112.78 (d, *J* = 25.6 Hz), 109.28 (d, *J* = 4.3 Hz), 106.98 (d, *J* = 23.8 Hz), 56.41. Anal. Calcd. for C_15_H_11_F_2_NO_3_S: C, 55.72; H, 3.43; N, 4.33. Found: C, 55.69; H, 3.45; N, 4.23.

5-chloro-1-((3-fluoro-4-methoxyphenyl)sulfonyl)-1*H*-indole (**2b**). Eluting system of petroleum ether: ethyl acetate (4:1). Beige-yellow solid, yield = 74.2%, m.p. = 121–122 °C. ^1^H-NMR (500 MHz, CDCl_3_) δ 7.89 (dt, *J* = 8.8, 0.7 Hz, 1 H), 7.65 (ddd, *J* = 8.7, 2.3, 1.3 Hz, 1 H), 7.56–7.54 (m, 1 H), 7.54–7.50 (m, 2 H), 7.28 (dd, *J* = 8.8, 2.1 Hz, 1 H), 6.96 (dd, *J* = 8.7, 7.8 Hz, 1 H), 6.61 (dd, *J* = 3.7, 0.8 Hz, 1 H), 3.89 (s, 3 H). ^13^C-NMR (126 MHz, CDCl_3_) δ 152.52 (d, *J* = 2.1 Hz), 151.56 (d, *J* = 265.6 Hz), 133.07, 131.98, 129.48 (d, *J* = 5.8 Hz), 129.34, 127.59, 124.98, 124.32 (d, *J* = 3.8 Hz), 121.08, 114.87 (d, *J* = 21.8 Hz), 114.46, 112.97 (d, *J* = 2.0 Hz), 108.79, 56.42. Anal. Calcd. for C_15_H_11_ClFNO_3_S: C, 53.03; H, 3.26; N, 4.12. Found: C, 53.11; H, 3.26; N, 4.21.

5-bromo-1-((3-fluoro-4-methoxyphenyl)sulfonyl)-1*H*-indole (**2c**). Eluting system of petroleum ether: ethyl acetate (8:1). Light yellow solid, yield = 74.2%, m.p. = 144–146 °C. ^1^H-NMR (500 MHz, CDCl_3_) δ 7.85 (d, *J* = 8.8 Hz, 1 H), 7.69–7.63 (m, 2 H), 7.57–7.50 (m, 2 H), 7.42 (dt, *J* = 8.9, 1.4 Hz, 1 H), 6.96 (t, *J* = 8.3 Hz, 1 H), 6.61 (d, *J* = 3.9 Hz, 1 H), 3.89 (s, 3 H). ^13^C-NMR (126 MHz, CDCl_3_) δ 152.22 (d, *J* = 1.9 Hz), 151.16 (d, *J* = 259.7 Hz), 133.43, 132.49, 127.53 (d, *J* = 24.8 Hz), 124.84, 124.32 (d, *J* = 3.9 Hz), 123.21, 116.96, 114.97, 114.84, 114.79, 112.98 (d, *J* = 2.2 Hz), 108.65, 56.43. Anal. Calcd. for C_15_H_11_BrFNO_3_S: C, 46.89; H, 2.89; N, 3.65. Found: C, 46.91; H, 2.92; N, 3.70.

#### 3.1.3. Synthesis of Intermediate Compound **2d**

To a stirred solution of 4-bromo-2-fluoroanisole (492 mg, 310 μL, 2.4 mmol) in dry toluene (4 mL), 5-chloroindole (303 mg, 2.0 mmol), rac-trans-N, N′-dimethylcyclohexane-1,2-diamine (57 mg, 63 μL, 0.4 mmol), and dry potassium phosphate tribasic (892 mg, 4.2 mmol) were added under a stream of nitrogen gas. Copper (I) iodide (19 mg, 0.1 mmol) was then added, and the mixture was refluxed for 24 h under an inert atmosphere. The reaction mixture was cooled at room temperature, diluted with 5 mL of dichloromethane, and filtered through a plug of silica gel. Additional dichloromethane (40–50 mL) was used to wash the silica, and the solvents were removed under reduced pressure. The resulting residue was purified by flash column chromatography.

5-chloro-1-(3-fluoro-4-methoxyphenyl)-1*H*-indole (**2d**). Eluting system of petroleum ether: ethyl acetate (15:1). White solid, yield = 71.8%, m.p. = 107–108 °C. ^1^H-NMR (500 MHz, CDCl_3_) δ 7.64 (d, *J* = 2.0 Hz, 1 H), 7.37 (d, *J* = 8.8 Hz, 1 H), 7.27 (d, *J* = 3.2 Hz, 1 H), 7.22 (dd, *J* = 11.5, 2.5 Hz, 1 H), 7.18 (ddt, *J* = 8.6, 6.1, 1.8 Hz, 2 H), 7.08 (t, *J* = 8.8 Hz, 1 H), 6.60 (d, *J* = 3.1 Hz, 1 H), 3.96 (s, 3 H). ^13^C-NMR (126 MHz, CDCl_3_) δ 152.36 (d, *J* = 248.7 Hz), 146.68 (d, *J* = 10.5 Hz), 134.48, 132.35 (d, *J* = 8.6 Hz), 130.07, 129.25, 126.01, 122.71, 120.30 (d, *J* = 3.7 Hz), 113.94 (d, *J* = 2.8 Hz), 113.15, 112.99, 111.25, 103.05, 56.55. Anal. Calcd. for C_15_H_11_ClFNO: C, 65.35; H, 4.02; N, 5.08. Found: C, 65.31; H, 4.12; N, 5.05.

#### 3.1.4. Synthesis of Final Compounds **3a**-**d**

The appropriate methyl ether derivative (2.6 mmol) was added to pyridine hydrochloride (810 mg, 7 mmol) that had been preheated at 210 °C for 10 min, and the mixture was stirred under a nitrogen atmosphere at 210 °C for 1 h. The reaction mixture was then poured onto crushed ice (40 mL), stirred at ambient temperature for 10 min, and the resulting mixture was extracted with ethyl acetate (3 × 20 mL). Combined organic phases were washed with brine, dried over anhydrous Na_2_SO_4_, filtered, and the solvent was removed under reduced pressure. The residue was purified by flash column chromatography.

4-((5-fluoro-1*H*-indol-1-yl)sulfonyl)-2-fluorophenol (**3a**). Eluting system of petroleum ether: ethyl acetate (3:1). Beige waxy semisolid, yield = 48.9%. ^1^H-NMR (500 MHz, DMSO-*d*_6_) δ 11.38 (s, 1 H), 7.93 (dd, *J* = 9.1, 4.4 Hz, 1 H), 7.84 (d, *J* = 3.5 Hz, 1 H), 7.82 (d, *J* = 2.3 Hz, 1 H), 7.66 (dd, *J* = 8.8, 2.4 Hz, 1 H), 7.41 (td, *J* = 9.8, 9.1, 2.6 Hz, 1 H), 7.17 (td, *J* = 9.2, 2.7 Hz, 1 H), 7.06 (t, *J* = 8.5 Hz, 1 H), 6.81 (d, *J* = 3.7 Hz, 1 H). ^13^C-NMR (126 MHz, DMSO-*d*_6_) δ 155.99 (d, *J* = 1082.7 Hz), 154.01 (d, *J* = 1104.0 Hz), 151.59, 132.03 (d, *J* = 10.8 Hz), 131.01, 129.33, 126.98 (d, *J* = 5.9 Hz), 125.34 (d, *J* = 3.1 Hz), 118.72 (d, *J* = 3.6 Hz), 115.87 (d, *J* = 21.6 Hz), 114.93 (d, *J* = 9.6 Hz), 112.93 (d, *J* = 25.7 Hz), 109.68 (d, *J* = 4.2 Hz), 107.48 (d, *J* = 24.0 Hz). Anal. Calcd. for C_14_H_9_F_2_NO_3_S: C, 54.37; H, 2.93; N, 4.53. Found: C, 54.31; H, 2.92; N, 4.50.

4-((5-chloro-1*H*-indol-1-yl)sulfonyl)-2-fluorophenol (**3b**). Eluting system of petroleum ether: ethyl acetate (4:1). Tan waxy semisolid, yield = 45.9%. ^1^H-NMR (500 MHz, DMSO-*d*6) δ 11.38 (s, 1 H), 7.93 (dd, *J* = 9.1, 4.4 Hz, 1 H), 7.84 (d, *J* = 3.5 Hz, 1 H), 7.82 (d, *J* = 2.3 Hz, 1 H), 7.66 (dd, *J* = 8.8, 2.4 Hz, 1 H), 7.41 (td, *J* = 9.8, 9.1, 2.6 Hz, 1 H), 7.17 (td, *J* = 9.2, 2.7 Hz, 1 H), 7.06 (t, *J* = 8.5 Hz, 1 H), 6.81 (d, *J* = 3.7 Hz, 1 H). ^13^C-NMR (126 MHz, DMSO-*d*_6_) δ 151.64 (d, *J* = 10.2 Hz), 150.70 (d, *J* = 268.5 Hz), 132.98, 132.33, 129.07, 128.48, 126.88 (d, *J* = 6.1 Hz), 125.39 (d, *J* = 3.2 Hz), 125.01, 121.47, 118.75 (d, *J* = 3.2 Hz), 115.91 (d, *J* = 21.6 Hz), 115.08, 109.21. Anal. Calcd. for C_14_H_9_ClFNO_3_S: C, 51.62; H, 2.79; N, 4.30. Found: C, 51.61; H, 2.79; N, 4.31.

4-((5-bromo-1*H*-indol-1-yl)sulfonyl)-2-fluorophenol (**3c**). Eluting system of petroleum ether: ethyl acetate (6:1). Reddish oil, yield = 60.3%. ^1^H-NMR (500 MHz, DMSO-*d*_6_) δ 11.42 (s, 1 H), 7.88 (dt, *J* = 8.8, 0.7 Hz, 1 H), 7.85 (d, *J* = 2.4 Hz, 1 H), 7.83 (d, *J* = 2.8 Hz, 2 H), 7.66 (ddd, *J* = 8.7, 2.4, 0.9 Hz, 1 H), 7.47 (dd, *J* = 8.8, 2.0 Hz, 1 H), 7.06 (t, *J* = 8.5 Hz, 1 H), 6.80 (dd, *J* = 3.7, 0.8 Hz, 1 H). ^13^C-NMR (126 MHz, CDCl_3_) δ 150.74 (d, *J* = 10.2 Hz), 149.79 (d, *J* = 268.5 Hz), 133.48, 131.53, 128.35, 127.20, 125.23 (d, *J* = 6.1 Hz), 124.75 (d, *J* = 3.2 Hz), 124.03, 120.89, 117.33 (d, *J* = 3.2 Hz), 114.98 (d, *J* = 21.6 Hz), 114.63, 108.48. Anal. Calcd. for C_14_H_9_BrFNO_3_S: C, 45.42; H, 2.45; N, 3.78. Found: C, 45.41; H, 2.49; N, 3.81.

4-(5-chloro-1H-indol-1-yl)-2-fluorophenol (**3d**). Eluting system of petroleum ether: ethyl acetate (15:1). Beige solid, yield = 77.9%, m.p. = 98-100 °C. ^1^H-NMR (500 MHz, DMSO-*d*_6_) δ 10.18 (s, 1 H), 7.66 (d, *J* = 2.1 Hz, 1 H), 7.61 (d, *J* = 3.2 Hz, 1 H), 7.44 (d, *J* = 8.8 Hz, 1 H), 7.41 (dd, *J* = 11.9, 2.6 Hz, 1 H), 7.19 (dd, *J* = 8.5, 2.2 Hz, 1 H), 7.15 (dd, *J* = 8.8, 2.2 Hz, 1 H), 7.11 (dd, *J* = 9.5, 8.6 Hz, 1 H), 6.62 (d, *J* = 3.2 Hz, 1 H). ^13^C-NMR (126 MHz, DMSO-*d*_6_) δ 151.27 (d, *J* = 243.1 Hz), 144.44 (d, *J* = 11.9 Hz), 134.38, 130.90, 130.54 (d, *J* = 8.6 Hz), 130.30, 125.04, 122.52, 121.07 (d, *J* = 3.0 Hz), 120.37, 118.64 (d, *J* = 3.8 Hz), 113.30 (d, *J* = 20.6 Hz), 112.29, 103.04. Anal. Calcd. for C_14_H_9_ClFNO: C, 64.26; H, 3.47; N, 5.35. Found: C, 64.30; H, 3.50; N, 5.35.

### 3.2. Biological Evaluation

#### 3.2.1. Animals

The ALR2 enzyme was isolated from rat lenses of the Fischer-344 series. Laboratory animal handling and experiment conduct was carried out in accordance with the Law on the Protection of Laboratory Animals (Hellenic Republic) and was declared to the Veterinary Authority of the Republic of Greece. The animal study protocol was approved by the Ethics Committee of the Prefecture of Central Macedonia (protocol code 270079/2500) for studies involving animals.

#### 3.2.2. Biological Reagents and Equipment

Reagents of di-sodium hydrogen phosphate dihydrate (Na_2_HPO_4_·2H_2_O), potassium dihydrogen phosphate anhydrous (KH_2_PO_4_), β-nicotinamide adenine dinucleotide 2′-phosphate reduced tetrasodium salt (NADPH·4Na^+^), DL-glyceraldehyde, ammonium sulfate anhydrous, ultrapure water, and dimethyl sulfoxide (DMSO) used for in vitro studies were supplied by Merck (Athens). Tissue homogenization was performed on a T25 digital ULTRA-TURRAX^®^ IKA (Janke & Kunkel-Str. 10 79219 Staufen, Germany) disperser. A Perkin Elmer Lambda 40 UV/VIS spectrophotometer was used for enzymatic assays.

#### 3.2.3. ALR2 Preparation

Eye lenses were quickly removed from Fischer-344 rats following euthanasia. The enzyme was partially purified by a well-established and previously reported procedure [50]. The lenses were removed from euthanized rats and were quickly stored at −80 °C until used. Great care was taken to clean the lenses mechanically from other ocular tissues and debris. The lenses were homogenized in 5 vol of cold ultrapure water (0.5 mL per lens), and the homogenate was centrifuged at 10,000 × rpm at 0–4 °C for 15 min. The supernatant was then collected, precipitated with a saturated solution of ammonium sulfate (40% saturation), and centrifuged at 10,000 × rpm at 0–4 °C for 15 min. Supernatant containing the partially purified ALR2 enzyme was used directly or stored at −80 °C for less than 24 h.

#### 3.2.4. ALR2 Enzymatic Assay

ALR2 activity was assayed spectrophotometrically by determining NADPH consumption (ε_340nm_ = 6300 M^−1^ × cm^−1^) [50]. The examined compounds were dissolved in DMSO (1% final concentration) at appropriate final concentrations (0.1–10 μM). The effects of the compounds on enzyme activity were determined by including the inhibitor in the reaction mixture at required concentrations. At the same concentration, the inhibitor was included in the reference blank. The reference blank contained all the following reagents except the substrate to correct NADPH oxidation, which is not associated with the substrate’s reduction. Briefly, 2.4 mL of 66.7 mM phosphate Na^+^/K^+^ assay buffer (pH = 6.2), 100 μL of 3.2 mM NADPH solution in assay buffer, 300 μL of enzyme supernatant, 90 μL of ultrapure water, and 10 μL of solution of tested inhibitors in DMSO in appropriate concentration or pure DMSO (served as control) were mixed in that order in a glass vial. Subsequently, 1 mL of that mixture was transferred to each 1.5 mL-UV quartz cuvette (10 mm path length), and 100 μL of assay buffer (reference blank) or 110 mM DL-glyceraldehyde solution (10 mM final concentration) in assay buffer was added to initiate the enzyme reaction. ALR2 activity was assayed by monitoring NADPH consumption (0.1 mM final concentration) at 340 nm for 5 min after incubating reaction mixtures for 1 min at 30 °C. Enzyme activity can be adjusted by diluting the enzyme preparation with ultrapure water so that an average reaction rate for control samples of 0.020 ± 0.005 absorbance units/min was achieved with a linear decrease in absorbance during a 5 min period. IC_50_ values were determined by least-squares regression analysis of the semi-logarithmic dose–inhibition curves. Each curve was generated using at least five compounds’ concentrations, causing inhibition from at least 15–80% in at least three triplicate repetitions.

#### 3.2.5. Docking Analysis

Three-dimensional compound structures were sketched in Discovery Studio 2020 (BIOVIA) software v21.1.0.20298 and then saved as Sybyl2 (mol2) files. The lowest energy conformations of all compounds were generated using the MMFF94 energy minimization protocol in the LigandScout v. 4.5 program [53]. Molecular docking simulations were performed on LigandScout v. 4.5 software by using AutoDock 4.2. The default docking settings were applied, and the binding affinity was calculated in kcal/mol. The lowest docking energy complex in terms of root mean square deviation value was then selected as the most favorable pose for each compound.

The crystal structure of human ALR2 in complex with novel sulfonyl-pyridazinone inhibitor CP-744809 (Protein Data Bank identification code: 1Z8A) [54] was used in the docking analysis. Although the in vitro inhibition assays of our compounds were conducted on rat ALR2, the use of the human ALR2 structure for docking is justified by the fact that the crystal structure of rat ALR2 co-crystallized with inhibitors is not yet available, and human and rat ALR2 sequences share 85% identity [53].

## 4. Results

### 4.1. Chemistry

**Figure 4:**
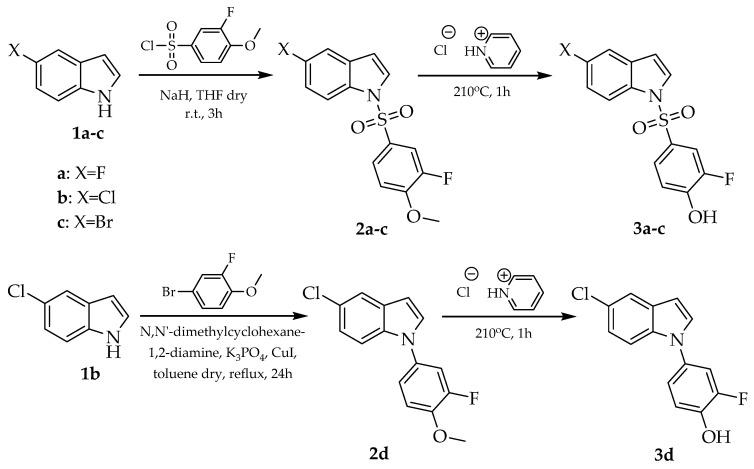
Synthesis of compounds **3a**-**d**.

The routes for synthesizing compounds **3a**-**d** are outlined in Figure 4 and followed according to the methods of Koutsopoulos et al. [50]. In the first step, 3-fluoro-4-methoxybenzenesulfonyl chloride reacted with appropriate 5-halogenated indoles **1a**-**c** in the presence of NaH in anhydrous tetrahydrofuran (THF) to provide N-sulfonyl substituted indole derivatives **2a**-**c**. Compound **2d** was prepared according to the methods of Papastavrou et al. [55] through a modified Ullmann-type reaction between 4-bromo-2-fluoroanisole and 5-chloroindole in dry toluene at reflux conditions using copper (I) iodide (5% mols) as a metal catalyst and rac-trans-*N*,*N*′-dimethylcyclohexane-1,2-diamine (20% mols) as a bidentate ligand. Subsequently, compounds **2a**-**d** were converted to the desired 2-fluorophenol derivatives **3a**-**d** through O-demethylation using pyridine hydrochloride at 210 °C for 1 h.

### 4.2. ALR2 Inhibition

We first performed an inhibition screening of the examined compounds at a cut-off concentration of 10 μM against our in-house, partially purified ALR2 enzyme from rat lenses. In this concentration of compounds, an encouraging inhibition mode was observed. Particularly, the 5-substituted indole derivatives of IIc with fluorine (**3a**), chlorine (**3b**), and bromine (**3c**) atoms demonstrated a 74.5 ± 2.6, 77.6 ± 1.3, and 80.6 ± 2.0% inhibition, respectively. In addition, compound **3d** displayed an inhibitory activity equal to 43.7 ± 1.3%.

**Figure 5:**
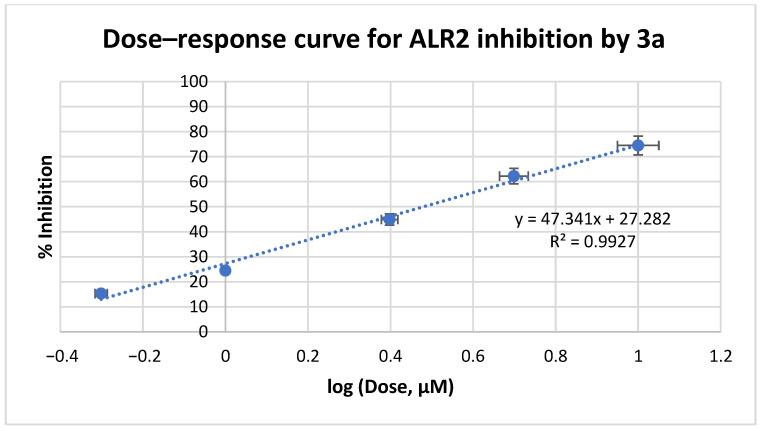
ALR2 inhibition assay curve for compound **3a**.

**Figure 6:**
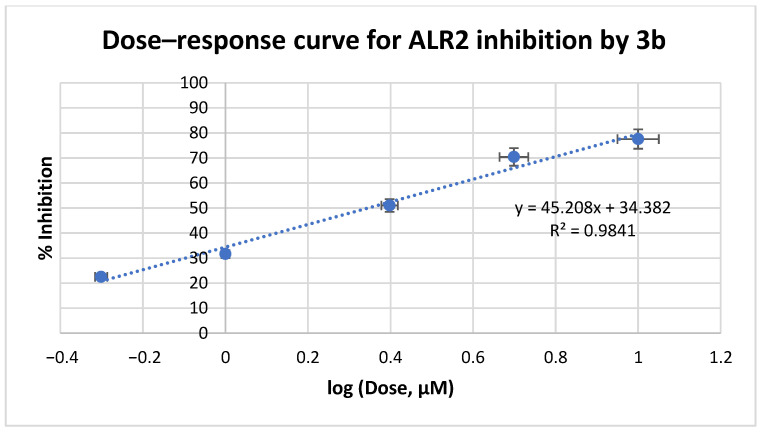
ALR2 inhibition assay curve for compound **3b**.

**Figure 7:**
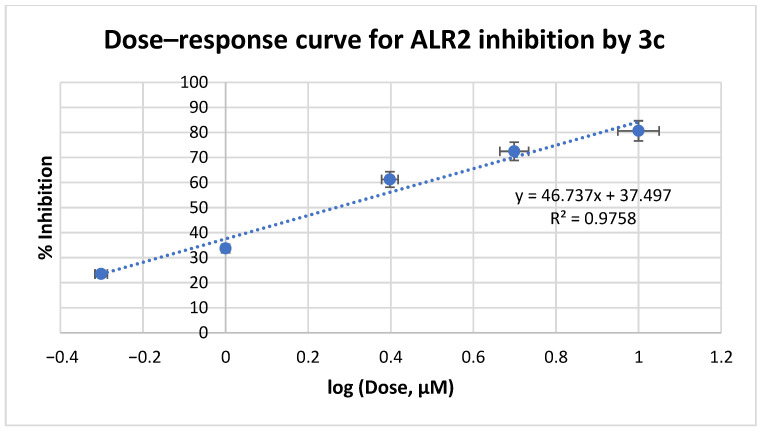
ALR2 inhibition assay curve for compound **3c**.

Based on these data, we further evaluated the IC_50_ values of **3a**-**c** at a range of 0.1–10 μM concentrations (Figure 5, Figure 6 and Figure 7, respectively). Inhibitory data expressed as the IC_50_ values ± Standard Deviation (SD) of all tested compounds are shown in Table 1. IC_50_ values of reference drugs, sorbinil and epalrestat, as well as the lead compound IIc were also displayed for comparison.

### 4.3. Docking Simulation

**Figure 8:**
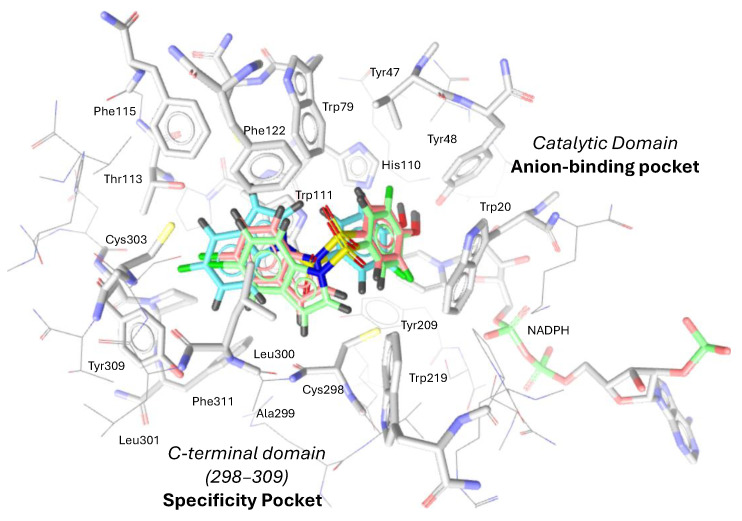
The binding mode in the crystal structure of AKR1B1 complexed with NADPH, according to molecular docking. Compounds **3a**, **3b**, and **3c** are illustrated in light blue, light green, and pink, respectively.

**Figure 9:**
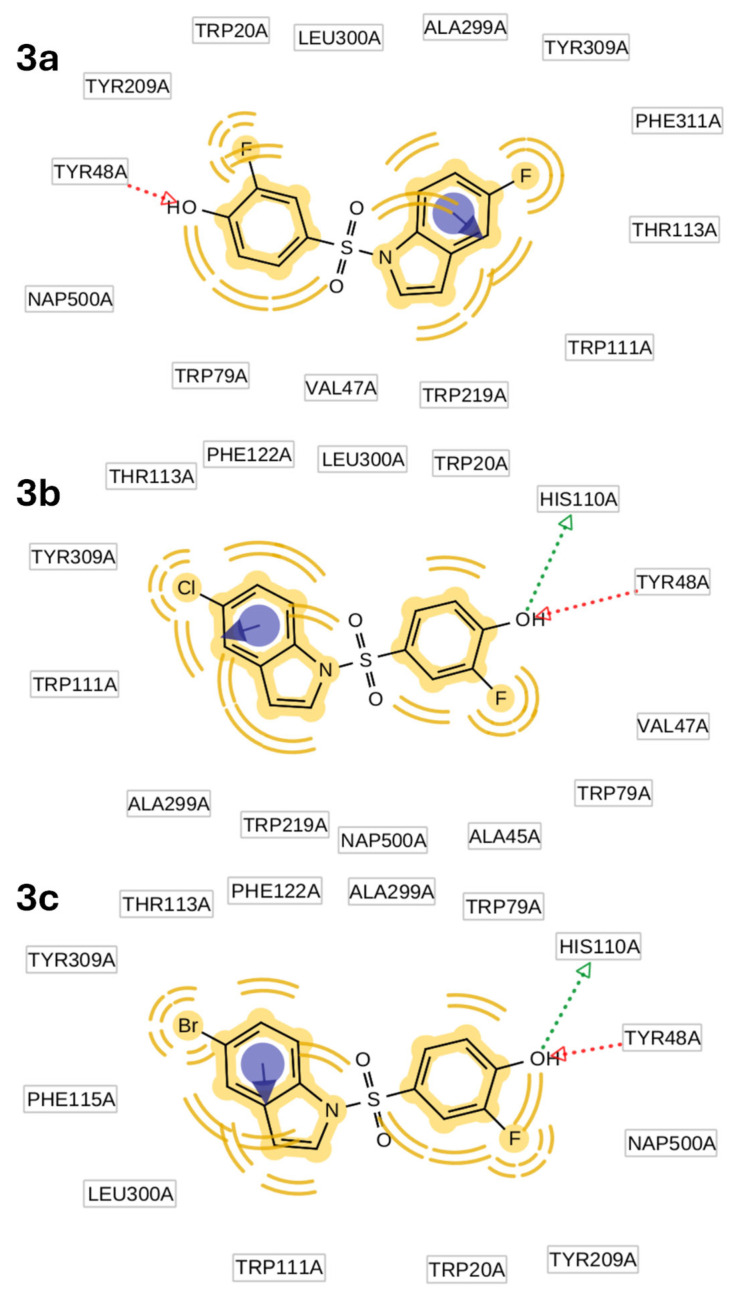
Docking simulation for **3a**-**c**. Hydrophobic, π-stacking, and hydrogen bonding donors and acceptors are highlighted in yellow, blue, green, and red arrows, respectively.

According to the docking simulation, all compounds adopt a common orientation upon binding to the active site, with the sulfonyl linker exposed to the solvent surface and aromatic rings buried into the key hydrophobic pockets (Figure 8). The free-binding docking energies of compounds **3a**-**c** were estimated at −9.19, −9.59, and −9.99 kcal per mol, respectively, thus meaning the complex formation between hALR2-**3c** and NADPH is the most favorable. In addition, Figure 9 illustrates interaction maps of compounds **3a**-**c** on the active site of AKR1B1. The analysis of these interactions is discussed in the following section.

## 5. Discussion

The biological evaluation of the compounds highlighted that the order of ALR2 inhibitory capacity in terms of IC_50_ follows **3c** > **3b**> **3a** > **3d**. According to the enzyme inhibition assay, it was also clear that the absence of the sulfonyl moiety significantly reduced the inhibitory capacity of compound **3d** against ALR2. This compound exhibited a poor inhibitory profile (43.7 ± 1.3%) at the cut-off concentration of 10 μM, whereas at a lower dose of 1 μM it was completely inactive. Therefore, we confirmed that the presence of this linker between the indole and o-fluorophenol aromatic rings is mandatory for the low micromolar inhibition of ALR2. Based on the data of Table 1, compounds **3a**-**c** are considered 12-, 9-, and 7.4-fold less strong ARIs than the reference equipotent drugs of sorbinil and epalrestat, which have been investigated under a similar in vitro assay protocol.

We also observed that the 5-substitution of the indole ring in our lead compound IIc with halogen atoms resulted in a slight decrease in the inhibitory power of compounds **3a**-**c**. Among halogens, the order of the increasing ALR2 inhibitory pattern follows **3a** (-F) < **3b** (-Cl) < **3c** (Br). Therefore, we can speculate that the halogen atom’s radius and electronegativity influence the enzyme inhibition. The increase in the size of the halogen atom enhances the intrinsic molecular lipophilicity and intensifies the halogen’s σ-hole interaction as the electron-withdrawing effect of the group decreases. These properties give rise to a plethora of hydrophobic interactions and halogen bonds that enable the binding of heavy halogen atoms (e.g., Br, I) within highly hydrophobic environments and enlarged active site pockets [57]. Among the 5-halogenated groups in the indole core, the bromine was the most tolerable substituent. Noticeably, the fluorine, chlorine, and bromine substitution of the aromatic scaffold led to a 3.1-, 2.3-, and 1.9-fold activity loss compared to the lead compound IIc against the rat ALR2 enzyme.

Although our strategy to optimize IIc with a strong inhibitory profile similar to that of epalrestat and sorbinil was not achieved, compounds **3a**-**c** are expected to exhibit a better in vivo tissue permeability and a more adequate pharmacokinetic profile than the ionized acetic acid derivative, epalrestat, and hydrophilic sorbinil reference ARI drugs, according to the physicochemical parameters of Table 1.

A docking analysis of compounds **3a**-**c** on the catalytic pocket of the human AKR1B1 crystal structure in the complex with the novel inhibitor CP-744809 (PDB ID: 1Z8A) was further performed to support and provide structural insight into the observed inhibitory data and visualize interactions between halogen atoms and active site residues in ALR2. For molecular docking studies, the crystal structure of human aldose reductase in the complex with this non-carboxylate sulfonyl-pyridazinone inhibitor was selected, as it better simulates the binding environment and interaction profile of our designed sulfonyl-containing compounds, in contrast to structures with ionized carboxylic acid ligands.

In all cases, the fluorophenyl moiety establishes hydrophobic interactions at distances of 4.0–4.5 Å (theoretical values = 3.5–5.0 Å) with lipophilic side chains of Trp20, Val47, Tyr48, Trp79, Trp111, and Tyr209 in the main binding domain, while the acidic hydroxyl group can participate in electrostatic contacts at distances of 3.0–3.2 Å (theoretical values = 2.8–4.0 Å) with the positively charged β-nicotinamide ring of NADP^+^ and hydrogen bonds at distances of 2.7–3.2 Å (theoretical values = 2.5–3.5 Å) with catalytic residues such as Tyr48 and/or His110 within the anion-binding pocket.

Hydrophobic contacts to the reduced β-nicotinamide ring in NADPH also play a crucial role in inhibiting ALR2. Moreover, the phenolic hydroxyl group in **3b** and **3c** establishes two hydrogen bonds with key residues of Tyr48 (as an acceptor with phenolic hydrogen) (O–H⋯O distance ≈ 2.7 Å) and His110 (as a donor with imidazole-nitrogen) (N⋯H–O distance ≈ 3.0 Å, responsible for aldehyde substrate reduction). Due to its distorted orientation, the absence of the latter type of interaction in **3a** may explain its capacity to block the ALR2 catalytic activity less significantly within this series.

Furthermore, we observed that the 5-substituted indole ring of **3a**-**c** efficiently penetrates the specificity pocket by establishing several hydrophobic interactions between the indole core and characteristic residues of Ala299, Leu300, and Phe122, which enable the “opening” of this cleft. In addition, hydrophobic contacts of the indole ring to Trp111 and Trp219 in the tryptophan-rich subcavity enhance the binding affinity of inhibitors. It is worth noting that the aforementioned interaction forces have also been estimated previously in the docking simulation of IIc.

Additional key pharmacophore features of strong ARIs, as in the case of IIc and CP-744809, are two parallel face-to-face π-stacking interactions between the aromatic ring (indole or benzofuran) and the side chain of Trp111. We observed that halogen atoms in the 5-halogenated indole moiety can be well anchored to key distinct residues in the selectivity pocket, albeit with a cost of driving the pyrrole moiety of the indole ring farther away from Trp111, thus losing an electrostatic attractive π-stacking interaction vital for potent ALR2 inhibition. Therefore, the indole ring in **3a**-**c** contributes only to staggered stacking (parallel displaced) (centroid–centroid distances ~4.1–4.4 Å) rather than face-to-face π-π interactions (typically distances of 3.5–4.5 Å) (Figure 9).

Halogen atoms are well buried in the selectivity pocket. Fluorine, chlorine, and bromine groups establish interesting hydrophobic attractive type contacts with the aromatic side chains of Trp111 and Tyr309, Phe311, or Phe155. A further interesting detail concerns the electrostatic polarization between the 5-halogen substituent at the indole moiety in the specificity pocket and the O^γ^ of Thr113. Although interactions between the O^γ^ of the latter residue and hydrogen or halogen bond groups have been lately recognized as a desirable target, none of the tested compounds form such connections. The 5-chloro and 5-bromo substituents at the indole moiety occupy approximately the same position as the 5-chloro group in the benzofuran ring in CP-744809 [54]. This fact suggests that similar polarization effects could be significant in the predicted **3b** and **3c** complexes with ALR2-NADPH, even though the σ-hole potential of simple aryl chlorides and bromides is poorer than their fluorinated counterparts and aryl iodides. According to the docking analysis, the O^γ^–X (X=Cl or Br) distances amount to greater than 3.5Å (particularly 4.0Å for Cl- and 3.7Å for Br- substituent, thus being unfavorable for halogen bond contacts). It should be mentioned that accepted limits for halogen bonding are typically 3.0–3.5 Å. The larger distance may be attributed to a non-optimal interaction geometry experienced by the halogen atoms in this position, given the restricted plasticity of the sulfonyl linker. On the contrary, bromine atoms in the crystal structures of known ARIs (Figure 1) (e.g., flexible 4-bromo-2-fluorobenzyl moiety) point directly forwards, whereas in the case of indolyl-sulfonyl-phenols, the heavy halogens are slightly off and orient towards the bottom of the pocket. Similarly, the 5-fluorine atom in the aromatic system cannot serve as a hydrogen bond acceptor for interactions with the hydroxyl moiety of Thr113, even though other aliphatic or aromatic fluorine atoms in ARIs, such as benzothiazoles (Figure 1), are excellent binders of the specificity pocket. We conclude that the 5-bromo substituent in **3c** can more efficiently approach Thr113 compared to other halogenated compounds (Br > Cl > F) through hydrophobic interactions, albeit without the occurrence of a the σ-hole electrostatic polarization on the O^γ^-side chain or hydrogen bonds. The same trend is highlighted among halogen atoms for targeting the adjacent Phe115, an ALR2-specific residue of great plasticity critical for the affinity of ARIs. Therefore, these interaction patterns can explain the descending order of the enzyme inhibition among compounds **3a**-**c**.

Even though these initial results suggest a trend, a broader substitution analysis is required to clarify whether bromine’s beneficial effect is due solely to its size or a combination of its size, volume, and hydrophobicity. Accordingly, additional compounds incorporating iodine, alkyl (e.g., methyl, trifluoromethyl, and tert-butyl), or even aryl (e.g., phenyl) groups are planned for synthesis to better define the steric and electronic requirements for optimal ALR2 inhibition.

## Figures and Tables

**Table 1:** Inhibitory data (IC_50_) of examined compounds **3a**-**d**, the lead compound IIc, and reference drugs against partially purified ALR2 from rat lenses, along with the acid dissociation constant (pKa) and the n-octanol/water partition coefficient at physiological plasma pH = 7.4 (logD_7.4_).

Compound	IC_50_ ± SD (μΜ)	pKa ^4^	logD_7.4_^4^
**3a**	3.02 ± 0.45	7.3 ± 0.8	2.48
**3b**	2.22 ± 0.28	7.3 ± 0.8	2.94
**3c**	1.85 ± 0.15	7.3 ± 0.8	3.23
**3d**	>10	9.2 ± 0.8	3.54
IIc	0.98 ± 0.16 ^1^	7.3 ± 0.8	2.29
sorbinil	0.25 ± 0.01 ^2^	8.3 ± 0.5	0.88
epalrestat	0.25 ± 0.02 ^3^	2.4 ± 0.8	−1.43

^1^ Koutsopoulos et al. [50], ^2^ Pegklidou et al. [56], ^3^ Kousaxidis et al. [51]. ^4^ In silico prediction in ADME Boxes v. 3.0, Pharma Algorithms, Inc., Toronto, ON, Canada.

## Data Availability

The raw data supporting the conclusions of this article will be made available by the authors on request.
